# Dual bilinear rotations

**DOI:** 10.5194/mr-7-89-2026

**Published:** 2026-06-24

**Authors:** Yannik T. Woordes, Burkhard Luy

**Affiliations:** 1 Institute of Organic Chemistry and Institute for Biological Interfaces 4 – Magnetic Resonance, Karlsruhe Institute of Technology (KIT), Hermann-von-Helmholtz-Platz 1, 76344 Eggenstein-Leopoldshafen, Germany

## Abstract

Bilinear rotations imply differing rotations on a spin I depending on the presence or absence of a bilinear coupling Hamiltonian in connection to a heteronucleus S . As such, spin system selective inversions using BIRD elements, excitations using TANGO, or general (effective) rotations using BANGO and/or BIG-BIRD, as well as multiplicity-edited rotations, are achievable. So far, the well-defined rotations were only imposed on a single spin, e.g., I , while the coupled heteronucleus experienced only an inversion or no rotation at all. Here, we introduce dual bilinear rotations that simultaneously allow spin system selective manipulations on both spins I and S as compared to the coupled spin system IS . Particularly with the advent of multi-receive experiments and/or super-sequences with the necessity of exciting and storing specific spin systems in a flexible way, this may open new possibilities in pulse sequence design. A general derivation of the approach is given, and a quadruple- J -resolved-type experiment for obtaining fully decoupled spectra optimized for different spin systems is introduced for demonstration.

## 1 Introduction

Bilinear rotations form important pulse sequence elements in NMR spectroscopy. The first element, called bilinear rotation, was introduced by Garbow, Weitekamp, and Pines in 1982 with the famous BIRD element (Garbow et al., 1982), which, with a variant producing the opposite proton inversion (Bax, 1983), has been summarized, extended, and systematically characterized by Uhrín, Liptaj, and Kövér (Uhrín et al., 1993). These elements were all based on the recognition that the number of coupled heterospins can be used to selectively invert and/or manipulate spins as first expressed in a multiplicity-editing-type experiment (Brown et al., 1981). After the BIRD element, more general bilinear rotations were also developed, like TANGO (Wimperis and Freeman, 1984), BANGO (Sørensen, 1994), or BIG-BIRD (Briand and Sørensen, 1997). Applications of bilinear rotations are manifold (Rutar, 1983; Bauer et al., 1984; Reynolds et al., 1989; Fehér et al., 2003; Lupulescu et al., 2012; Castañar and Parella, 2015; Schulze-Sünninghausen et al., 2017; Bodor et al., 2020; Nagy et al., 2021; Bigler et al., 2024; Schulze-Sünninghausen et al., 2026). Recent fundamental extensions of the technique involve the application to isotope-labeled samples via band-selective refocusing on the X nucleus (BASEREX) (Haller et al., 2019; Bodor et al., 2020; Sebák et al., 2022) and to improved robustness against variations in couplings, offsets, and B_1_ inhomogeneities in the so-called COB-BIRD (Woordes et al., 2025) and generally in COB and COB3 bilinear rotations (Woordes and Luy, 2026).

All bilinear rotations reported so far concern the defined treatment of spin I , usually ^1^H, while the S spin experiences a spin inversion, spin refocusing, or no effective rotation. While finishing the last mentioned paper, we became aware that bilinear rotations may also work independently on both involved spins. Furthermore, we added the well-known fact that the multiplicity of involved spins follows a very general and easy rule for the basic bilinear rotation elements, leaving us with an interest in generally applying bilinear rotations for I spins, as well as for S spins. In this article, we look into the possibility to combine such elements even further into dual bilinear rotations that act as two concurrent independent bilinear rotations on either of the coupled spins. We give a short theoretical foundation for a general construction scheme including complex bilinear rotations like Dual-BANGO-BIG-BIRD and give an experimental example with a fast-pulsing quadruple- J -resolved-type pulse sequence with, all together, four dual bilinear rotations; this follows the NORD (no relaxation delay) principle (Nagy et al., 2021; Koos and Luy, 2019; Sørensen, 2024) and allows the detection of all four spectra without interscan relaxation delays and simultaneous detection of both protons and the heteronucleus.

## 2 The general structure of bilinear rotations

Bilinear rotations are spin-system-selective heteronuclear building blocks that distinguish spins I , which are not directly coupled to a heteronucleus, from I spins in a spin system I{S} , where the spin I is coupled to a spin S via a large heteronuclear coupling J . In all basic bilinear rotation elements, the difference between uncoupled and coupled spins is induced by a transverse π rotation with different phases for the I and I{S} spin systems. The BIRD element with its variants has two flanking 90° pulses and acts as a spin-system-selective 180° pulse, which is used in a very wide range of applications, including spectral cleanup (Kurz et al., 1991; Schmieder et al., 1991), homonuclear decoupling for obtaining pure shift spectra (Lupulescu et al., 2012; Sakhaii et al., 2009; Kiraly et al., 2015; Aguilar et al., 2011; Donovan and Frydman, 2015; Haller et al., 2022; Gyöngyösi et al., 2021; Saurí et al., 2015; Saurí et al., 2017; Kaltschnee et al., 2014), enhanced coupling determination (Fehér et al., 2003; Reinsperger and Luy, 2014; Schulze-Sünninghausen et al., 2017; Timári et al., 2014; Timári et al., 2016), and enhanced resolution in a J -evolved dimension (Furrer et al., 2007), to name just a few. The different types of BIRD sequences are well characterized by the d,r,X nomenclature for *directly* bound protons, *remote* protons, and the heteronucleus X . A BIRD^*d*,*X*^ element, in this case, inverts direct protons and the heteronucleus X , while remote protons are left unchanged.

TANGO bilinear rotations provide a 90° (or arbitrary β -) pulse for one type and either 0 or 180° for the other type of I and I{S} spin systems. To clearly specify the type of rotations produced by a specific TANGO sequence, we would like to introduce a nomenclature in analogy to BIRD: a TANGO^*d*,*X*^-(90°)^*r*^ element will then describe a TANGO sequence where directly bound protons and the heteronucleus X will be inverted, while the remote protons will experience a 90° rotation. We will use this notation later for specifying specific TANGO elements.

BANGO, as the third type of basic bilinear rotation, allows rotations about any flip angles $\beta^{I}$ and $\beta^{I\{S\}}$ for the spin systems. Both TANGO and BANGO are applied for spin-system-selective excitations, for example, in X-filtering-type experiments (Rance et al., 1987; Berger, 1989; Poppe et al., 1993). Finally, the excitation element BIG-BIRD rotates initial $I_{z}$ polarization into any final position that can be reached by effective $\beta_{\phi^{I}}^{I}$ and $\beta_{\phi^{I\{S\}}}^{I\{S\}}$ rotations, introducing the effective phases $\phi^{I}$ and $\phi^{I\{S\}}$ for the two spin systems. This type of spin-system-selective element has recently found particular use with the advent of super-sequences and the NORD (no relaxation delay) or generalized Ernst angle approach (Nagy et al., 2021; Koos and Luy, 2019; Sørensen, 2024).

**Figure 1:**
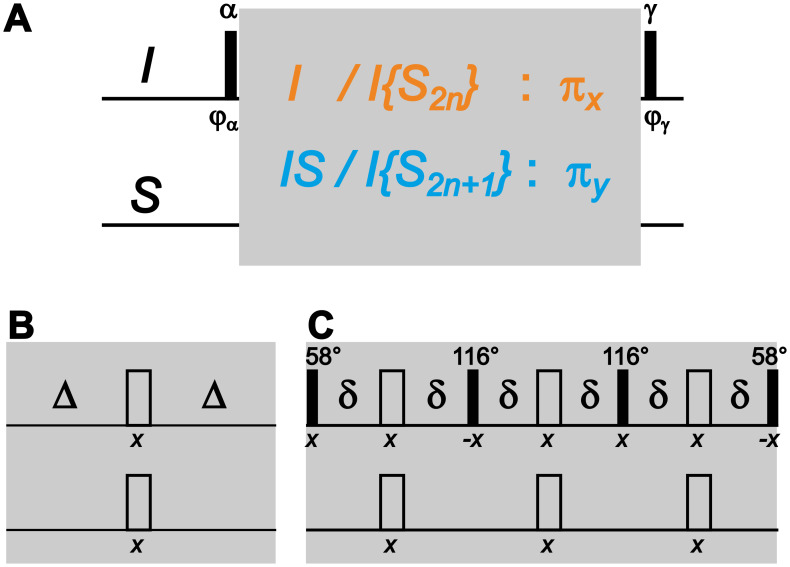
General structure of bilinear rotations. BIRD, TANGO, BANGO, and BIG-BIRD all consist of a spin-system-selective π rotation with flanking pulses $\alpha_{\varphi_{\alpha }}$ and $\gamma_{\varphi_{\gamma }}$ , where only the latter define the specific type of bilinear rotation **(A)**. The central π rotation element resulting in an effective $\pi_{x}$ rotation for I spins with an even number of directly coupled S spins and in an effective $\pi_{y}$ rotation for I spins with an odd number of directly coupled S spins can be achieved by the original refocused delay with Δ=1/(2J) **(B)** or more sophisticated π rotation elements like the one used in the COB-BIRD (Woordes et al., 2025; Woordes and Luy, 2026) with delays δ= 2.583 ms for the coverage of a J -coupling range of 120–250 Hz **(C)**.

As shown in Woordes and Luy (2026) and mentioned above, the central refocused delay of overall duration 1/ J provides the distinction of the two spin systems and is common to all basic bilinear rotation elements (see Fig. 1), while flanking pulses define the different effects of the bilinear rotation elements. The central refocused delay, as shown in Fig. 1, provides a $\pi_{x}$ rotation for the uncoupled I spin and a $\pi_{y}$ rotation for the I spin of an I{S} spin system if the delay is matched to the heteronuclear coupling Δ=1/J . It is thereby important to note that the full rotational properties of all three Cartesian components are being used and that the difference in phase for the uncoupled and coupled spin systems applies equally to the S spin for an uncoupled spin S or the coupled {I}S spin system. Flanking pulses applied on the I spin in conventional bilinear rotations generally affect only the I spins, and S spins experience a 180° rotation or no effective rotation if an additional 180° pulse on the S spin is applied at the end of the refocused delay period.

Using this common construction principle of all bilinear rotations, it has been shown previously that it is sufficient to make the refocused delay robust to significantly enhance all different elements. As such, the central blocks derived in the COB-BIRD (Woordes et al., 2025) can be used directly to make any type of basic bilinear rotation robust against coupling variations in the J -coupling range of 120–250 Hz (Fig. 1C). It is important to note that a full universal rotation element should be used that rotates all magnetization components in the desired way. More simple inversion elements that only invert the z component spins like in the original JC-BIRD (Garbow et al., 1982) will not be applicable in general. Full universal π rotations, on the other hand, will work independently of the applied nuclei. As such, the sequence shown in Fig. 1C may also be applied with pulses on I and S spins exchanged.

Using coupling-compensated pulse sandwiches for the individual 180° pulses, like the J -compensated BUBI (Ehni and Luy, 2013) and BUBU (Ehni et al., 2022) pulse sandwiches, and, potentially, additional offset-compensated universal rotation pulses for other flip angles, all bilinear rotation elements can also be made robust for larger offset ranges.

## 3 Dual bilinear rotations

We now have a closer look at the transformations. The central π rotation element produces a $\pi_{x}$ rotation for both isolated I and S spins, but single-spin magnetization of coupled spins matching the condition Δ=1/2J , represented by either I{S} or S{I} , lead to an effective propagator $U=\mathrm{exp}(-i\pi 2I_{y}S_{y})$ and therefore to effective $\pi_{y}$ rotations on all spins of the coupled spin systems. Bilinear operators with components of both spins and annotated with IS perform accordingly. For the ideal case with perfectly matched couplings and perfect pulses, all resulting rotations can be summarized as

$$\begin{array}{lccc} I: & I_{x}\to I_{x}; & I_{y}\to -I_{y}; & I_{z}\to -I_{z}\\ S: & S_{x}\to S_{x}; & S_{y}\to -S_{y}; & S_{z}\to -S_{z}\\ I\{S\}: & I_{x}\to -I_{x}; & I_{y}\to I_{y}; & I_{z}\to -I_{z}\\ S\{I\}: & S_{x}\to -S_{x}; & S_{y}\to S_{y}; & S_{z}\to -S_{z}\\ IS: & 2I_{x}S_{x}\to 2I_{x}S_{x}; & 2I_{y}S_{x}\to -2I_{y}S_{x}; & 2I_{z}S_{x}\to 2I_{z}S_{x}\\ & 2I_{x}S_{y}\to -2I_{x}S_{y}; & 2I_{y}S_{y}\to 2I_{y}S_{y}; & 2I_{z}S_{y}\to -2I_{z}S_{y}\\ & 2I_{x}S_{z}\to 2I_{x}S_{z}; & 2I_{y}S_{z}\to -2I_{y}S_{z}; & 2I_{z}S_{z}\to 2I_{z}S_{z}. \end{array}.$$

**Figure 2:**
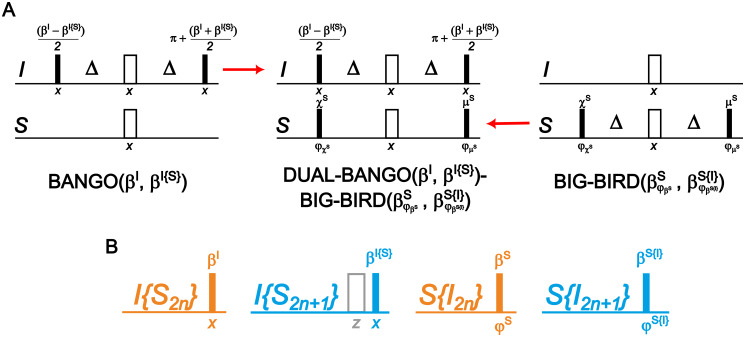
Construction of a dual bilinear rotation using the example of a general dual BANGO-BIG-BIRD. The I -spin part of a BANGO sequence with specific effective rotations $\beta^{I}$ and $\beta^{I\{S\}}$ is combined with the S -spin part of a BIG-BIRD sequence for specific point-to-point transformations described by effective flip angles and phases $\beta_{\varphi^{S}}^{S}$ and $\beta_{\varphi^{S\{I\}}}^{S\{I\}}$ to obtain the overall dual-BANGO( $\beta^{I}$ , $\beta^{I\{S\}}$ )-BIG-BIRD( $\beta_{\varphi^{S}}^{S}$ , $\beta_{\varphi^{S\{I\}}}^{S\{I\}}$ ) sequence **(A)**. The resulting effective rotations are shown in **(B)**, where the open gray box represents an effective phase shift by 180°.

As mentioned already in the previous section, all effective rotations of the central π rotation element are identical for I and S spins due to symmetry. More so, I spins and S spins evolve completely independently and can also do so simultaneously. As a result, flanking pulses of the original bilinear rotations that, so far, always focused on the I spin effective rotations may equally and even simultaneously be applied to the S spin. Consequently, the most general universal rotations following the BANGO principle can be applied with the four individually defined flip angles $\beta^{I}$ and $\beta^{S}$ and $\beta^{I\{S\}}$ and $\beta^{S\{I\}}$ . Equally, BIRD-, TANGO-, and BIG-BIRD-type bilinear rotations can be applied and mixed simultaneously for the two spins. The general construction principle is visualized in Fig. 2 for a dual-BANGO-BIG-BIRD bilinear rotation generated from a BANGO element for I spins and a BIG-BIRD element for S spins.

While, in conventional bilinear rotations, the heterospin is either left untouched or inverted, any dual bilinear rotation, of course, applies defined rotations on both spins I and S . This has to be taken into account in corresponding pulse sequences, especially if bilinear coherences are present and need to be controlled.

Another property of any bilinear rotation concerns more complex spin systems compared to just the two-spin system discussed so far. The central refocusing element of bilinear rotations is also used as a building block for multiplicity editing in attached proton test (APT) (Brown et al., 1981; Patt and Shoolery, 1982; Torres et al., 1990; Torres et al., 1993; Bigler et al., 2024) and ME-HSQC (Kay and Bax, 1989; Davis, 1991; Zhang and Wang, 1991; Schmieder et al., 1991; Willker et al., 1993) experiments, as is also included in Fig. 1. Coherence transfers derived for uncoupled spins I or S therefore also apply for any even multiplicity $I\{S_{2n}\}$ and $S\{I_{2n}\}$ with integer n=0,1,2,… , and coupling-matched coherence transfer in IS spin systems also applies to odd multiplicity $I\{S_{2n+1}\}$ and $S\{I_{2n+1}\}$ spin systems. This property of basic bilinear rotations is maintained in dual bilinear rotations and will be used in a demonstration experiment in the following section.

## 4 Experimental demonstration

We were looking for experimental verification of the dual-BIRD principle and came up with a particular J -resolved super-sequence that separates ^13^C-bound protons from other protons and quaternary carbons (Cq) and CH_2_ groups from CH and CH_3_ groups in a single 2D experiment. The sequence shows the principal benefit, a viable scheme for implementation with basic cleanup, but also the shortfall in the case of non-negligible homonuclear coupling evolution during the bilinear rotations.

**Figure 3:**
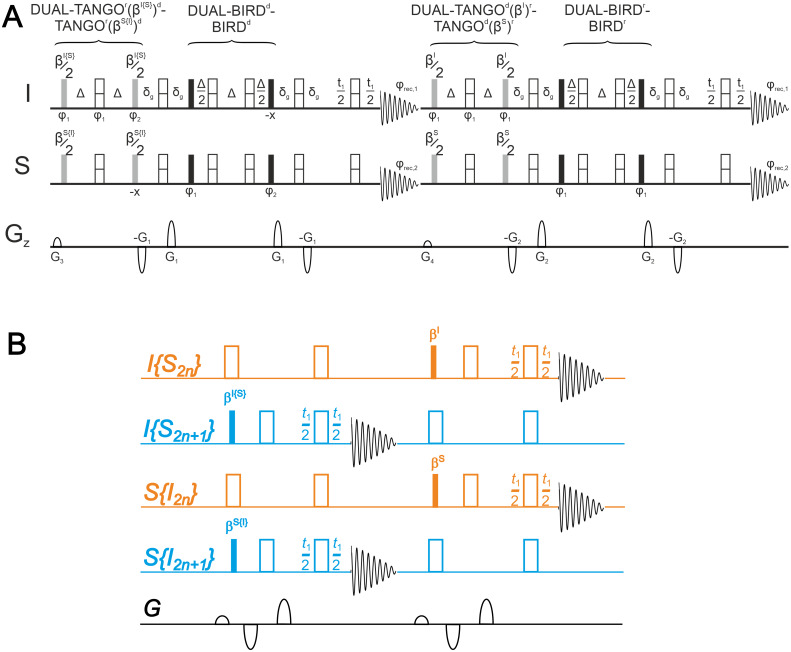
Quadruple J -resolved experiment designed to rapidly acquire four heteronuclear and homonuclear decoupled spectra for differentiating $I\{S_{2n}\}$ , $I\{S_{2n+1}\}$ , $S\{I_{2n}\}$ , and $S\{I_{2n+1}\}$ with, typically, $I{=}^{1}$ H and $S{=}^{13}$ C. The actual pulse sequence **(A)** and a simplified pseudo-sequence for the various differentiated spin systems **(B)** are given. ** (A)** Solid black bars describe hard 90° pulses, while solid gray bars stand for hard pulses with flip angles as annotated. Open bars with a dividing central line describe short universal rotation 180° pulses, as given in the Supplement, that are able to cover the relatively narrow chemical shift ranges of glucose. Delays Δ are matched to the heteronuclear coupling between IS spins according to 1/( 2J ). The delays with duration $\delta_{g}$ are determined by corresponding gradient durations and necessary gradient recovery delays. Gradients of 250 µ s duration and a recovery delay of 50 µ s have been used on our spectrometer with typical strengths of G_1_ = 81 %, G_2_ = 79 %, G_3_ = 29 %, and G_4_ = 19 % of the maximum gradient strength of the probe head ( ≈ 50 G cm^−1^). A basic phase cycle has been applied with $\varphi_{1}$ = x , y , -x , -y , $\varphi_{2}$ = -x , -y , x , y , $\varphi_{\mathrm{rec},1}$ = x , y , -x , -y , and $\varphi_{\mathrm{rec},2}$ = x , -x . Please note that the sequence requires dual-receive capabilities. The corresponding pulse sequence with COB-enhanced dual bilinear rotations is given in the Supplement. **(B)** The pseudo-sequence summarizes the effective pulses of all bilinear rotations for the four different spin system classes that are differentiated by the dual bilinear rotations of the quadruple J -resolved experiment. The DUAL-TANGO blocks result in either β excitation or a polarization inversion, while the DUAL-BIRD blocks result in either 180° pulses or no effective rotation. In addition, bipolar gradients are summarized as single gradients for further simplification. Altogether, for each nucleus in both acquisition schemes, a single spin system is selectively excited, with the BIRD filters being selectively refocused for spectral cleanup and finally J -evolved on both nuclei simultaneously with all homonuclear and heteronuclear couplings prior to acquisition. Ernst angle excitation can be achieved selectively for each class of spin systems by choice of the corresponding TANGO sequence. Unused magnetization is stored along z before acquisition.

The sequence consists of, all together, four dual bilinear rotations, two excitation elements, and two G-BIRD-type refocusing elements for basic cleanup. The resulting super-sequence is shown in Fig. 3. The Dual-TANGO^*r*^-( $\beta^{I\{S\}}$ )^*d*^-TANGO^*r*^-( $\beta^{S\{I\}}$ )^*d*^ for potential Ernst-angle-type excitation is followed by a dual-BIRD^*d*^-BIRD^*d*^ element with surrounding, refocused gradients and a J -evolution period on both channels before the first dual-receive acquisition period. The dual TANGO, in this case, excites protons bound to ^13^C with the specific excitation angle $\beta^{I\{S\}}$ and, at the same time, excites carbons with a single or three directly attached protons by $\beta^{S\{I\}}$ , while all other proton and carbon spins experience an inversion. The dual-BIRD^*d*^ element, on the other hand, refocuses all spins with a direct ^1^H, ^13^C coupling, while all transverse magnetization of remote spins is dephased by the surrounding gradients. During the J -evolution period with chemical shift refocusing on both nuclei, as well as during acquisition, all homonuclear and heteronuclear couplings evolve to the well-known 45° tilted pattern of conventional homonuclear J -resolved spectra.

In the second part of the experiment, a dual-TANGO^*d*^-( $\beta^{I}$ )^*r*^-TANGO^*d*^-( $\beta^{S}$ )^*r*^ element ensures Ernst-angle-type excitation for all remote spins, i.e., non-^13^C-bound protons and carbons in Cq and CH_2_ groups. Equally, the excited nuclei are refocused by the following dual-BIRD^*r*^-BIRD^*r*^ element, while all other transverse magnetization is dephased by the surrounding gradients. J -evolution and acquisition periods are identical to the first part of the super-sequence. In order to remove unwanted magnetization leftovers from previous scans, additional gradients were applied before each of the two parts.

The sequence was tested on a sample readily available in our laboratory, uniformly ^13^C-labeled glucose dissolved in DMSO- $d_{6}$ . For determination of Ernst angles, we measured maximum $T_{1}$ times for the different nuclear species, resulting in ≈ 1.2 s (OH), ≈ 650 ms (^1^H{^13^C}), ≈ 400 ms (^13^C{^1^H}), and ≈ 230 ms (^13^C{^1^H_2_}). Using only the last acquisition time of 350 ms as the repetition time, Ernst angles would result in 41.7, 54.3, 65.4, and 77.4°, respectively. Taking the full repetition time including all switching and transfer delays with maximum indirect J -evolution time, 1.43 s, results in 72.3, 83.6, 88.4, and 89.9°, respectively, where the latter three may also be approximated by 90° without noticeable loss in sensitivity. An experimental screening of flip angles, surprisingly, gave the best results for Ernst angles calculated from the full repetition time. We therefore chose $\beta^{I}=$ 72.3° and $\beta^{I\{S\}}=\beta^{S\{I\}}=\beta^{S}=90$ ° for the spectra shown.

**Figure 4:**
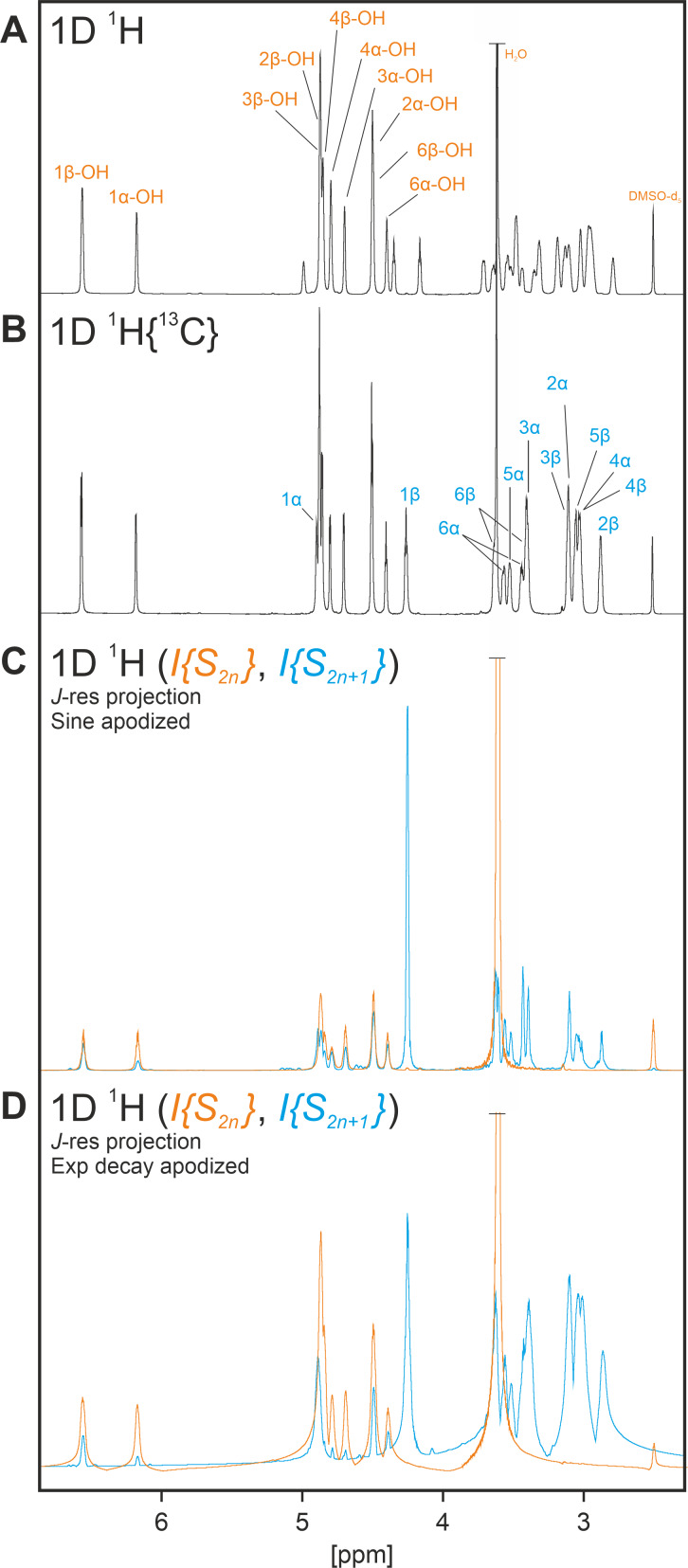
Various ^1^H 1D spectra acquired on uniformly ^13^C-labeled glucose dissolved in DMSO- $d_{6}$ . **(A)** A conventional, fully coupled 1D spectrum; **(B)** a carbon heteronuclear decoupled 1D spectrum; and **(C)** two homonuclear and heteronuclear decoupled, spin-system-selective 1D spectra obtained from the quadruple- J -resolved experiment described in Fig. 3. **(D)** The same as **(C)** but with different apodization. In orange, the subspectrum of non-^13^C-bound protons is displayed, which, for the glucose sample, comprises all hydroxyl groups, H_2_O, and residual partly protonated DMSO- $d_{5}$ . Corresponding assignments are provided in **(A)**. The blue subspectrum is designed to mainly contain directly ^13^C-bound protons, for which the assignments are given in **(B)**. For the spin-system-selective experiments, parameters were set as follows: Δ=1/(2⋅144Hz) , and $\beta^{I}=$ 72.3°, $\beta^{I\{S\}}=\beta^{S\{I\}}=\beta^{S}=90$ ° (see text for reasoning); ^1^H pulses with 90° duration of 9.7 µ s were irradiated at 4.48 ppm, and ^13^C pulses with 90° duration of 12.0 µ s were irradiated at 80.0 ppm; acquisition times were 250 ms in the indirect $t_{1}$ dimensions using 64 increments each; direct acquisition times with 4096 complex data points were 350 ms in all cases. Spectra were zero-filled to 128 × 8192 points. The 2D J -resolved-type spectra were processed either using sine apodization in both dimensions for absorptive type line shapes **(C)** or using exponential apodization in both dimensions for more reliable peak intensities **(D)**. The 2D J -spectra were tilted and projected to obtain the 1D spectra shown. Corresponding 2D spectra are shown in the Supplement.

Fully coupled and heteronuclear decoupled proton spectra of the sample are shown in Fig. 4A and B, with corresponding assignments of exchanging (A) and ^13^C-bound protons (B). The super-sequence, on the other hand, was applied, and individual spectra were separated and processed as described in the figure caption of Fig. 4, with projections of the selective J -spectra shown in (C). The orange spectrum containing only protons without ^13^C attached displays a very clean selection, with only hydroxyl groups, water, and unlabeled DMSO- $d_{5}$ being visible. The blue spectrum, however, has a multitude of signals containing the desired homo-decoupled signals of ^13^C-bound protons but also significant peaks from other protons, which have up to half the intensity of the desired singlets. The situation is particularly severe for the desired H1 α , which is next to the artifact signal of equal intensity originating from the two overlapping signals 2 β and 3 β -OH. The main reason for significant artifact signals, reduced H1 α , and a very intense H1 β signal is the sine-apodization, which has been applied to ensure sharp, absorptive-like line shapes. The apodization minimizes the H1 α signal due to its zero crossing at the center of $t_{1}$ with its ≈ 4 Hz coupling to H2 α , while it maximizes all other signals with no or only large couplings like for the H1 β signal. With different apodization, such as the multiplication with an exponential decay function, signal intensities are more equally distributed, with a clearer suppression of unselected signals (see Fig. 4D). The multitude of homonuclear and long-range heteronuclear couplings generally lead to reduced performance of the bilinear rotation elements, which are designed for spin systems without such couplings, but the effect on the ^1^H spectra is relatively small. Transfer elements are, furthermore, compromised by chemical exchange of the hydroxyl groups and second-order artefacts, like in the case of 2/3/4/5β protons with particularly reduced signal intensities.

**Figure 5:**
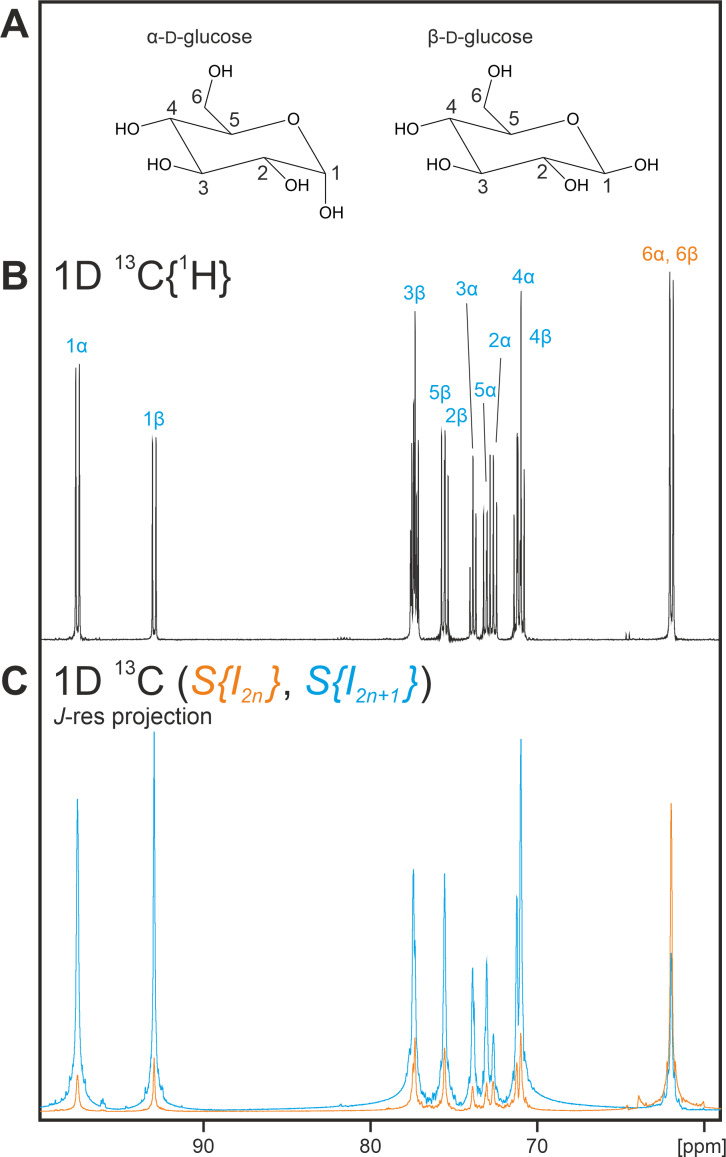
Various ^13^C 1D spectra acquired on uniformly ^13^C-labeled glucose **(A)** dissolved in DMSO- $d_{6}$ . **(B)** A proton heteronuclear decoupled 1D spectrum. **(C)** Two homonuclear and heteronuclear decoupled, spin-system-selective 1D spectra obtained from the quadruple- J -resolved experiment described in Fig. 3. In orange, the subspectrum optimized for $S\{I_{2n}\}$ , i.e., CH_2_ groups, is displayed. The blue subspectrum is optimized for carbons in $S\{I_{2n+1}\}$ spin systems, which is reduced to CH groups in glucose. Due to strong homonuclear ^13^C, ^13^C couplings, the spin system selectivity during bilinear rotations is severely reduced to a slight preference in intensities. The different spin systems, however, can be identified by the relative intensities of the two spectra. For the spin-system-selective experiments, parameters were set as follows: Δ=1/(2⋅144Hz) ; $\beta^{I}=$ 72.3°, and $\beta^{I\{S\}}=\beta^{S\{I\}}=\beta^{S}=90$ ° (see text for reasoning); ^1^H pulses with 90° duration of 9.7 µ s were irradiated at 4.48 ppm, and ^13^C pulses with 90° duration of 12.0 µ s were irradiated at 80.0 ppm; acquisition times were 250 ms in the indirect $t_{1}$ dimensions using 64 increments each; direct acquisition times with 4k complex data points were 350 ms in all cases. Spectra were zero-filled to 128 × 8k points. The 2D J -resolved-type spectra were processed using sine apodization in the indirect dimension and exponential apodization in the directly detected dimension. Subsequently, spectra were tilted and projected to obtain the 1D spectra shown. The 2D spectra are shown in the Supplement.

The equivalent ^13^C spectra are shown in Fig. 5. The decoupled 1D experiment shows the multitude of ^13^C–^13^C couplings that are decoupled in the homo-decoupled projections of the 45° tilted J -spectra of the super-sequence. The multiplicity selection of the two subspectra in Fig. 5 unfortunately does not work properly as all signals are present in the subspectra with significant intensities. Only the relative intensities allow a distinction of CH and CH_2_ groups. While overlapping C6 α/β values show a more intense signal in the orange spectrum for even multiplicities, all other signals are more intense in the blue spectrum for odd multiplicities. The reason for the low selectivity of the carbon spectra lies in the large ^13^C, ^13^C multiplets that span multiplet widths of up to 80 Hz. With heteronuclear one-bond couplings on the order of 140 Hz, the distinction of multiplicities during bilinear rotations is, in this case, quite poor, with transfer via the homonuclear couplings being on a similar order as the heteronuclear coupling. It is actually quite positive that the distinction of multiplicities based on the relative intensities is still possible in all cases.

As the super-sequence allows the detection of four spectra in a single experiment, the overall detection of corresponding spectra in individual experiments lasts about 4 times longer. Corresponding spectra from individual experiments are shown in the Supplement.

The super-sequence of Fig. 3 can also be run using the compensated COB and COB3 bilinear rotations introduced in Woordes et al. (2025) and Woordes and Luy (2026), respectively. Resulting spectra, in this case, look very similar to the ones shown in Figs. 4 and 5, but artefacts are even stronger due to the longer duration of the compensated sequences that allows longer evolution times of homonuclear couplings and exchange to occur. The sample used, glucose, comprises only small ranges of ^1^H and ^13^C chemical shifts, and, also, ${}^{1}J_{\mathrm{CH}}$ couplings are relatively uniform so that no improvement to the classical bilinear rotations is expected if the compensated sequences are applied. This will change for other samples with large chemical shift ranges and significantly varying coupling constants. The corresponding super-sequences with COB-based bilinear rotation, together with resulting spectra for the glucose sample, are given in the Supplement.

**Figure 6:**
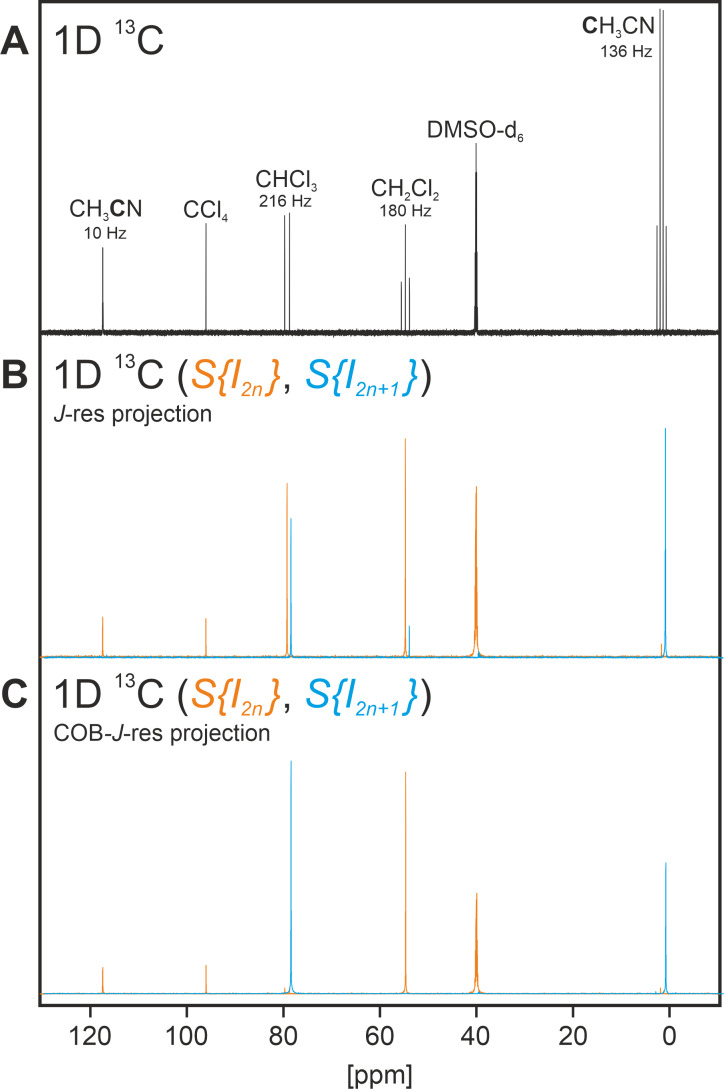
The ^13^C 1D **(A)** and ^13^C projections of the quadruple- J -resolved spectra **(B)** and the COB-enhanced quadruple- J -resolved spectra **(C)** for a mixture of tetrachlorocarbon, chloroform, dichloromethane, and acetonitrile dissolved in DMSO- $d_{6}$ . The assignment of the different components is provided in **(A)** together with applying one-bond coupling constants. The ^13^C subspectra for groups with an even number of attached protons are displayed in orange, and subspectra for carbons with an odd number of attached protons are displayed in blue in **(B)** and **(C)**. The blue subspectra are slightly shifted to avoid overlap of individual signals. Corresponding 2D J -spectra are shown in the Supplement.

As suggested by the reviewers, we performed additional experiments to demonstrate the performance of the sequence on the ^13^C side in a case without significant distortions due to ^13^C, ^13^C couplings. We therefore used a sample readily available in our laboratory containing tetrachlorocarbon, chloroform, dichloromethane, and acetonitrile dissolved in DMSO- $d_{6}$ at natural abundance isotope levels. In this case, no ^13^C, ^13^C couplings are present, and the quality of selection of the different multiplicities is solely determined by the properties of the dual bilinear rotations used. A corresponding fully coupled 1D-^13^C and two ^13^C projections of the conventional quadruple- J -resolved experiment and the COB-enhanced version of the quadruple- J -resolved experiment (referred to as COB- J -res) are shown in Fig. 6. Clearly, spectra are nicely decoupled (with the exception of the deuterate solvent), and, in orange spectra, in all cases, $S\{I_{2n}\}$ spin systems (i.e., C and CH_2_ groups) should show the larger intensities, while $S\{I_{2n+1}\}$ spin systems (i.e., CH and CH_3_ groups) should dominate in the blue spectra. Chloroform, however, has a one-bond coupling of 216 Hz in DMSO and is not covered by the bandwidth of conventional bilinear rotations, and, also, the CH_2_ group of dichloromethane deviates from the ideal coupling range, leading to severe deviations in expected spectra. The J -compensated COB-enhanced bilinear rotations, instead, cover all one-bond couplings in the range of approximately 120–260 Hz, and corresponding COB- J -res projections accordingly show very clear selections with very few residual artifact signals, just as expected.

## 5 Discussion and conclusion

Understanding that any basic bilinear rotation element can be applied to both I and S spins simultaneously without interference, the dual bilinear rotation principle is easily derived. It represents a generalization of bilinear rotations and might become of interest for dual-detection experiments, acquiring both I and S spins simultaneously. We also foresee a particular interest for quantum-computing-type applications, where the overall state of a spin systems needs to be manipulated in a spin-system-dependent way.

The dual principle can be used to combine any two bilinear rotation elements, i.e., BIRD, TANGO, BANGO, and BIG-BIRD elements, to be applied to the I and S spins simultaneously. The approach should also work with bilinear rotation modifications, like CAGEBIRD (Koskela et al., 2004) or BASEREX (Haller et al., 2019) for homonuclear J -distortion suppression and band selectivity, respectively.

The dual approach can be applied straightforwardly to all single-spin coherences on both I and S spins. Special care, however, has to be taken for the application to bilinear operators like $2I_{x}S_{z}$ , to pick an arbitrary example. In such cases, unexpected outcomes may result as the two spins will be rotated individually, as if $I_{x}$ and $S_{z}$ would be present as independent linear operators, only multiplied again to become the bilinear operator after the dual bilinear rotation element. As such, a conventional BIRD^*d*,*X*^ applied to $2I_{x}S_{z}$ , for example, would result in $2(-I_{x})(-S_{z})=2I_{x}S_{z}$ . A conventional TANGO^*X*^(270°)^*d*^ (resulting in 270° on the *directly* X -attached spin, 0° on the *remote* spin, and 180° on the heteronucleus X ) applied to S as the direct or remote spin, on the other hand, would result in $2(-I_{x})(S_{y})=-2I_{x}S_{y}$ . A dual BIRD^*d*,*X*^-TANGO^*X*^(90°)^*d*^, finally, will lead to transfers $I_{x}\to -I_{x}$ and $S_{z}\to S_{y}$ and, overall, to $2I_{x}S_{z}\to -2I_{x}S_{y}$ . This needs to be taken into account if dual bilinear rotations are applied.

As long as the condition for universal $\pi_{x}$ rotations for isolated I and S spins and universal $\pi_{y}$ rotations for all spins in IS spin systems is simultaneously fulfilled, any central transfer element, including the COB and COB3 elements derived in Woordes et al. (2025) and Woordes and Luy (2026), respectively, is also applicable for dual bilinear rotations.

## Supplement

10.5194/mr-7-89-2026-supplement The supplement related to this article is available online at https://doi.org/10.5194/mr-7-89-2026-supplement.

## Data Availability

Spectra in JCAMP-DX and Bruker format, together with Bruker pulse programs used for acquisition of example NMR spectra, are available at 10.35097/6d6mmwg567q7hku7 (Woordes and Luy, 2026).
