# Supplementary material for: Dual bilinear rotations
Source: Magn Reson (Gott). 2026 Jun 24;7(1):89–98. doi: 10.5194/mr-7-89-2026 (PMC13375272; doi:10.5194/mr-7-89-2026)
Supplement: The supplement related to this article is available online at https://doi.org/10.5194/mr-7-89-2026-supplement. [file mr-7-89-2026-supplement.pdf]

Supplement of Magn. Reson., 7, 89–98, 2026
https://doi.org/10.5194/mr-7-89-2026-supplement
© Author(s) 2026. CC BY 4.0 License.

Open Access
MAGNETIC
RESONANCE

Supplement of

Dual bilinear rotations

Yannik T. Woordes and Burkhard Luy

Correspondence to: Burkhard Luy (burkhard.luy@kit.edu)

The copyright of individual parts of the supplement might differ from the article licence.

Contents

S1 The COB-variant of the quadruple J-resolved pulse sequence 2

S2 Spectra of the COB-variant of the quadruple J-resolved experiment for glucose 3

S3 2D J-resolved subspectra for glucose 4

S4 Proton subspectra of the quadruple J-resolved experiment for mixture 6

S5 2D J-resolved subspectra for mixture 7

S6 Broadband refocusing pulse shape used (^{13}C) 9

S7 Broadband refocusing pulse shape used (^{1}H) 13

S1 The COB-variant of the quadruple J-resolved pulse sequence

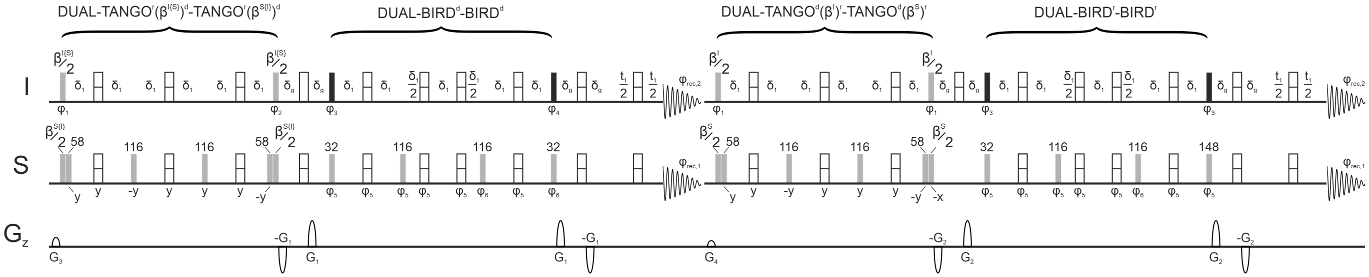

Figure S1. Quadruple J-resolved experiment designed to rapidly acquire four hetero- and homonuclear decoupled spectra for differentiating $I\{S_{2n}\}$, $I\{S_{2n+1}\}$, $S\{I_{2n}\}$, and $S\{I_{2n+1}\}$ employing the coupling compensated bilinear rotations as described in (Woordes et al., 2025) with typically I = ^{1}H and S = ^{13}C. Black, solid bars describe hard 90°pulses, while gray solid bars stand for hard pulses with flip angles as annotated. Open bars with a dividing central line describe universal rotation 180°pulses. Delays δ_1 correspond to 2.583 ms. The delays with duration δ_g are determined by corresponding gradient durations and necessary gradient recovery delays. Gradients of 250 μs duration and a recovery delay of 50 μs have been used on our spectrometer with typical strengths of G_1 = 81%, G_2 = 79%, G_3 = 29%, and G_4 = 19% of the maximum gradient strength of the probehead (≈ 50 G/cm). Phases of the pulses are all x unless indicated otherwise. A basic phase cycle has been applied with φ_1 = x, $-x$, φ_2 = $-x$, x, φ_3 = x, $-x$, y, $-y$, φ_4 = $-x$, x, $-y$, y, φ_5 = x, y, $-x$, $-y$, φ_6 = $-x$, $-y$, x, y, $\varphi_{rec,1}$ = x, $-x$, $-x$, x, and $\varphi_{rec,2}$ = x, $-x$. Please note that the sequence requires dual receive capabilities.

S2 Spectra of the COB-variant of the quadruple J-resolved experiment for glucose

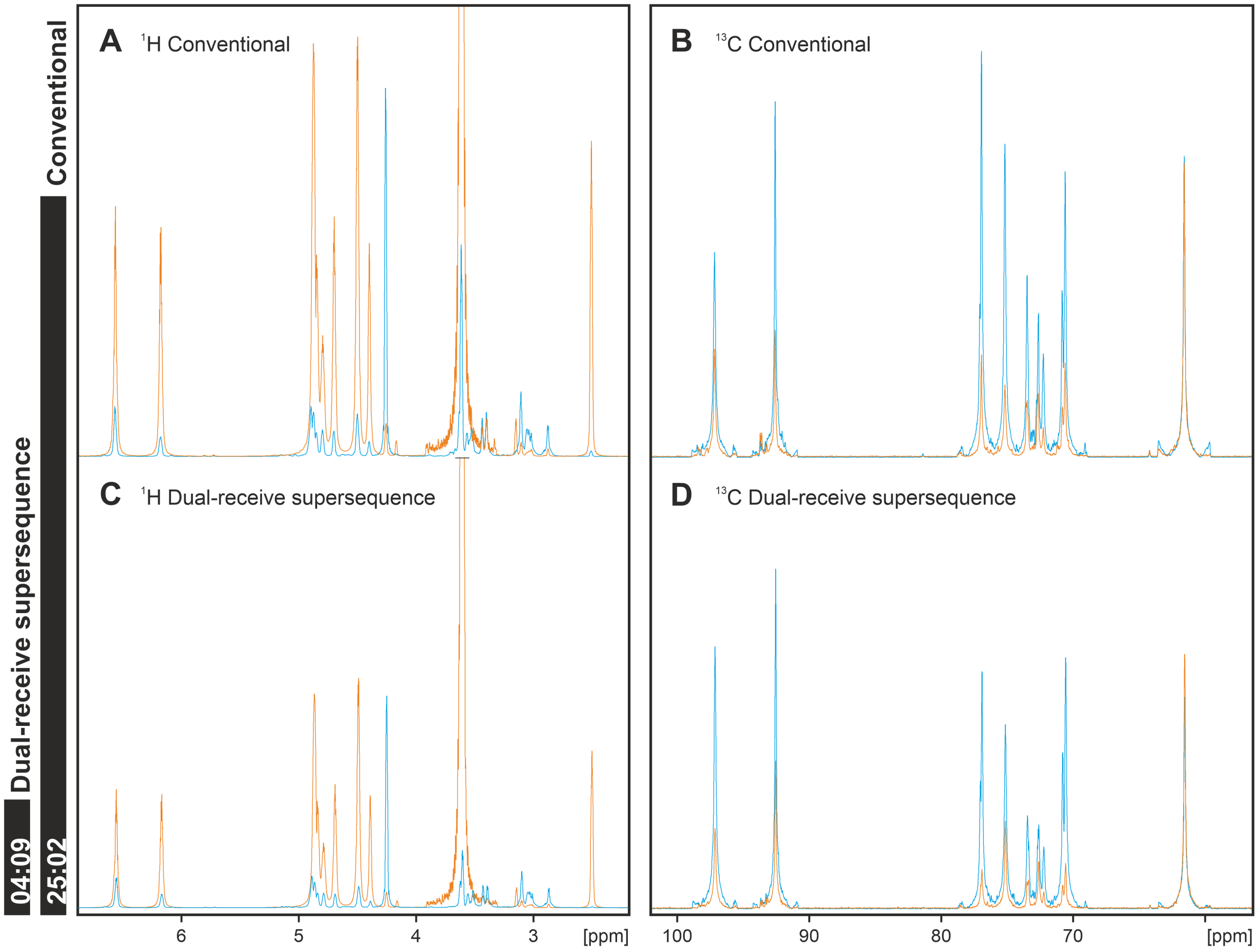

Figure S2. Various fully heteronuclear and homonuclear decoupled ^{1}H (A, C) and ^{13}C (B, D) 1D spectra on uniformly ^{13}C labeled glucose dissolved in DMSO-d_6. All spectra are obtained via 2D J-resolved experiments either in the conventional matter where all four need to be measured seperately (A, B), or in a quadrupole J-resolved experiment as described in Fig. S1. In the conventional case the total experiment time amounts to 25 minutes and 2 seconds, whereas the implementation of dual-receiver with NORD type interleaving reduces the time to just 4 minutes and 9 seconds, as highlighted in the bars on left of the figure. The orange and blue color indicate $I\{S_{2n}\}$ / $S\{I_{2n}\}$, and $I\{S_{2n+1}\}$ / $S\{I_{2n+1}\}$ selection. Assignment can be found in the main text figures 4 and 5. β^{I} = $\beta^{I\{S\}}$ = $\beta^{S\{I\}}$ = β^{S} = 90°; ^{1}H pulses with 90°duration of 9.7 μs were irradiated at 4.48 ppm, ^{13}C pulses with 90°duration of 12.0 μs at 80.0 ppm; acquisition times were 250 ms in the indirect t_1 dimensions using 64 increments each; direct acquisition times with 4k complex data points were 350 ms for ^{1}H and 160 ms for ^{13}C. Spectra were zero-filled to 128×8k points. 2D J-resolved type spectra were processed using sine-apodization in the direct dimension and either sine- or exponential-apodization in the direct dimension for the ^{1}H and ^{13}C spectra, respectively. Subsequently, the 2D spectra were tilted and the projection along the direct dimension is shown.

S3 2D *J*-resolved subspectra for glucose

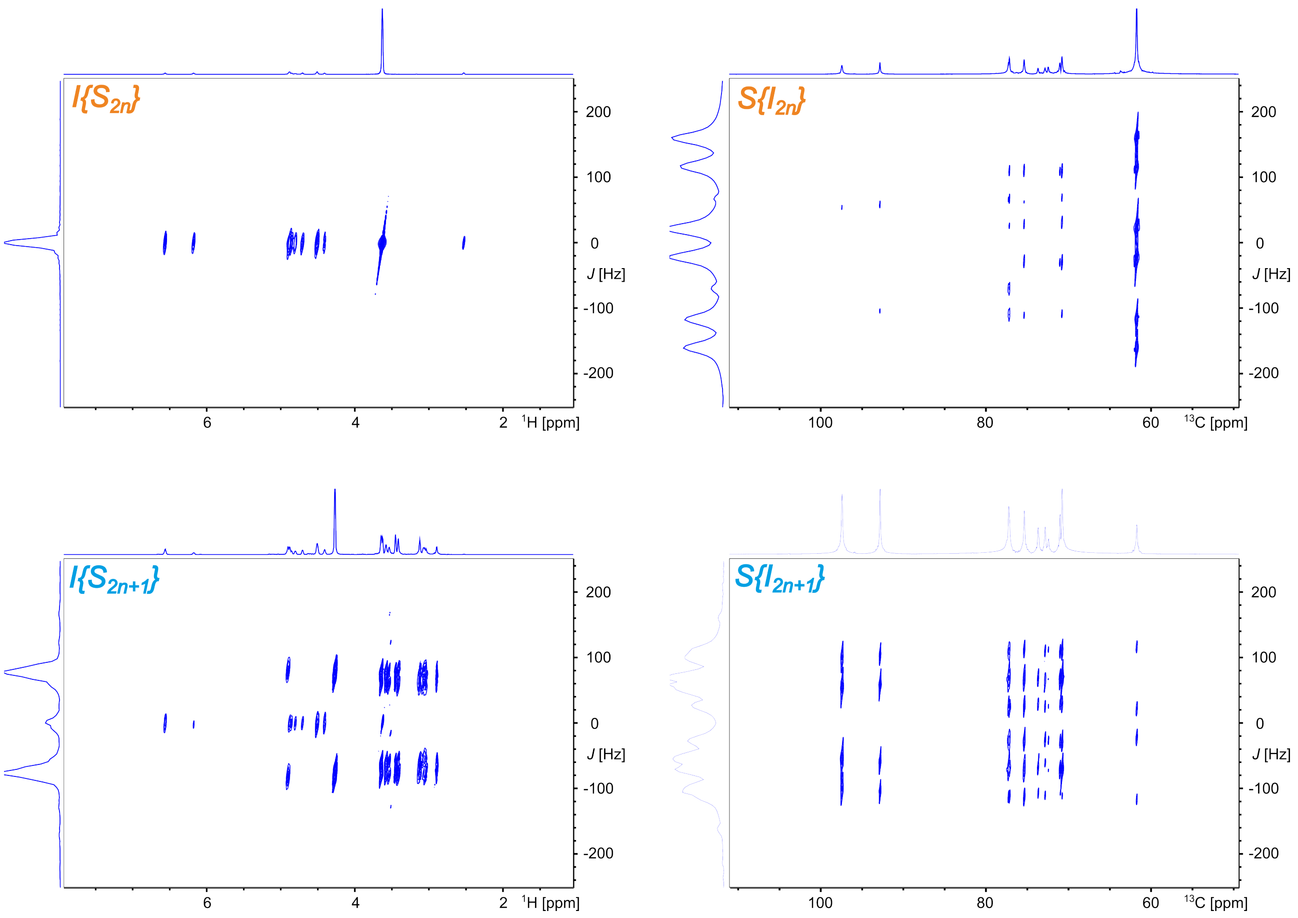

Figure S3. 2D *J*-subspectra for the projections shown in Figs. 4 and 5 in the main text. top left) non-^{13}C-bound protons; bottom left) ^{13}C-bound protons; top right) ^{13}C spectrum of carbons with an even number of attached protons; bottom right) ^{13}C spectrum of carbons with an odd number of attached protons.

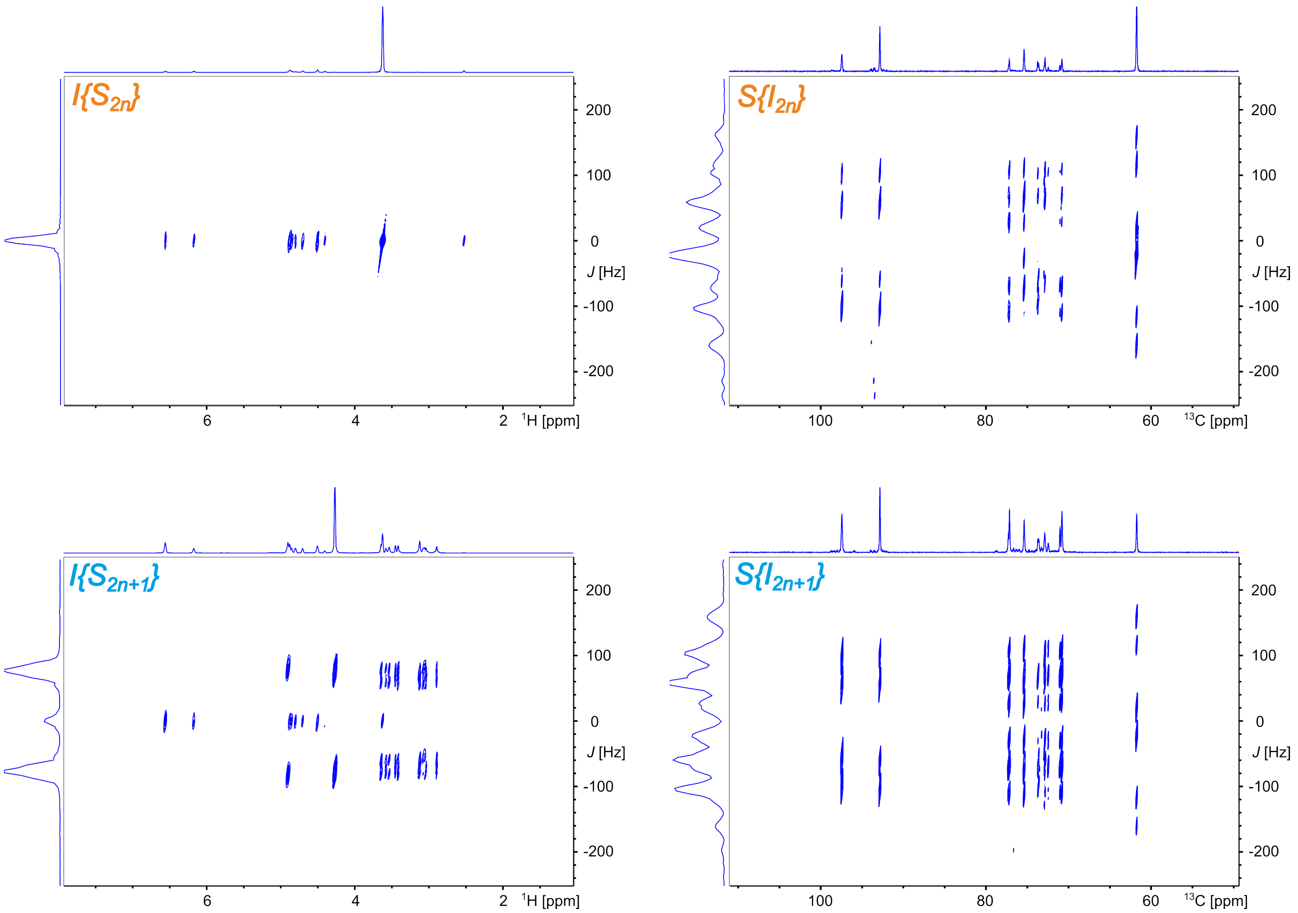

Figure S4. 2D J-subspectra for the COB-enhanced version of D-glucose. top left) non-^{13}C-bound protons; bottom left) ^{13}C-bound protons; top right) ^{13}C spectrum of carbons with an even number of attached protons; bottom right) ^{13}C spectrum of carbons with an odd number of attached protons.

S4 Proton subspectra of the quadruple J-resolved experiment for mixture

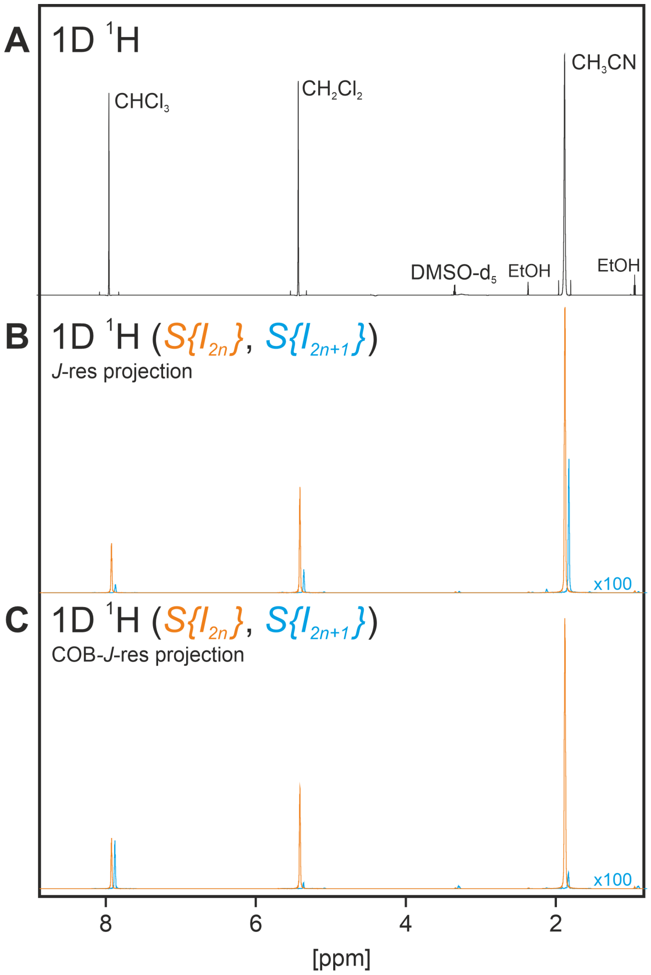

Figure S5. In Fig. 6 of the main text, ^{13}C subspectra of the quadruple J-resolved experiment are shown for a mixture of four small molecule compounds dissolved in DMSO-d_6. The proton counterparts are summarized here. Please be aware that non-^{13}C-bound protons form the majority for the natural abundance sample. The opposite experiment leads to a very weak spectrum of natural abundance ^{13}C-bound protons. No additional information is gained, as, of course, all molecules have ^{12}C and ^{13}C bound protons. Signal intensities of the blue spectra may also be compromised by residual ^{12}C bound proton artefacts.

S5 2D J-resolved subspectra for mixture

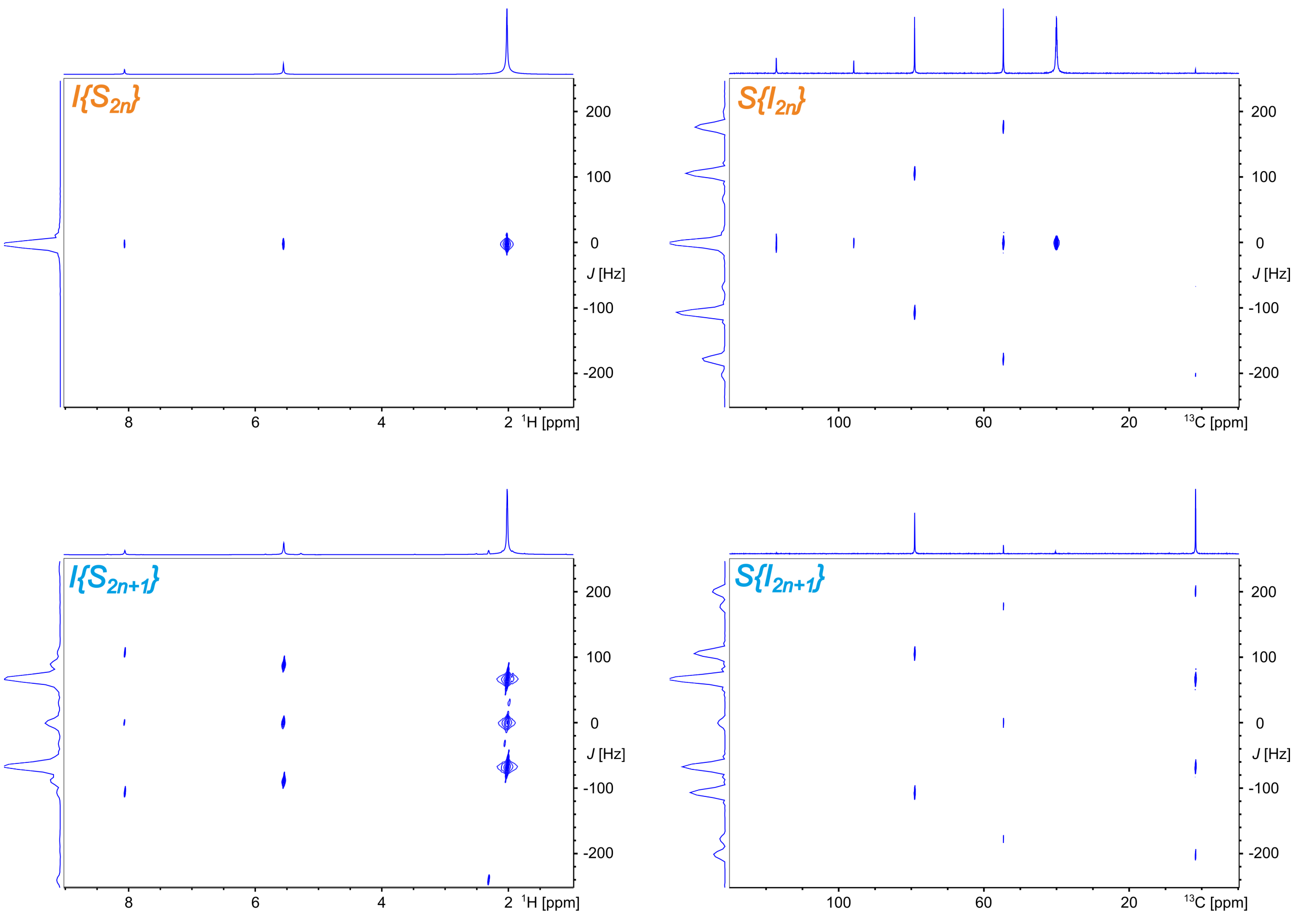

Figure S6. 2D J-subspectra for the projections shown in Fig. 6 and S5. top left) non-^{13}C-bound protons; bottom left) ^{13}C-bound protons; top right) ^{13}C spectrum of carbons with an even number of attached protons; bottom right) ^{13}C spectrum of carbons with an odd number of attached protons.

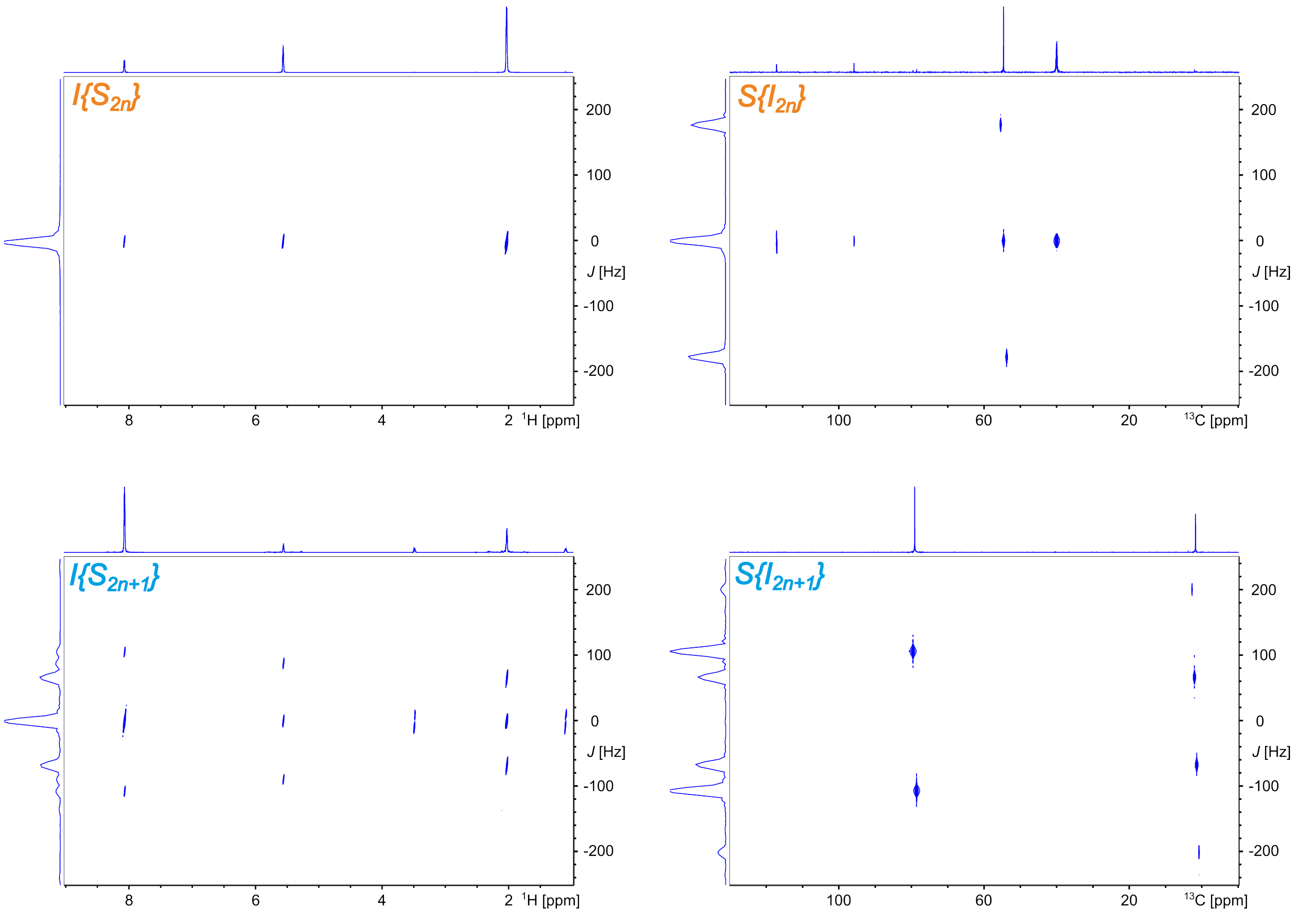

Figure S7. 2D J-subspectra for the COB-enhanced version of the mixture described in the main text. top left) non-^{13}C-bound protons; bottom left) ^{13}C-bound protons; top right) ^{13}C spectrum of carbons with an even number of attached protons; bottom right) ^{13}C spectrum of carbons with an odd number of attached protons.

S6 Broadband refocusing pulse shape used (^{13}C)

Broadband universal rotation 180°pulse shape (BURBOP-180) for a bandwidth of 10 kHz, an rf-amplitude ν_{rf} = 15 kHz, B_1-field compensation of ±10%, and a pulse length t_p of 300 μs in Bruker Topspin shape format (amplitude, phase). Quality factor of the pulse shape was >0.99999 . Please note that it is *not* J-compensated like the BUBI (Ehni and Luy, 2013) or BUBU (Ehni et al., 2022) pulse sandwiches. The pulses, however, are very short and unwanted effects within the bilinear rotations will be small. To save space, we present it in two-column format.

```
##TITLE= BURBOP180x_300us_BW10k_RF15k_pm10_99999.brk
##JCAMP-DX=
##DATA TYPE= Shape Data
##OWNER= yannik t. woordes and burkhard luy
##DATE= 2025-10-14
##Max_Amplitude= 15000.0
##PULSE_LENGTH= 300.0 us
##NPOINTS= 300
##XYPOINTS= (XY..XY)
100.0, -2.905323\\
100.0, -8.488571
100.0, -13.146732
100.0, -17.22671
100.0, -20.93722
100.0, -24.457521
100.0, -27.933281
100.0, -31.482645
100.0, -35.219082
100.0, -39.30336
100.0, -44.091457
99.999998, -49.906011
99.995691, -57.517012
100.0, -68.040283
100.0, -81.747596
99.997975, -98.738144
99.998345, -115.912561
100.0, -127.961477
99.999999, -135.060486
100.0, -139.051867
100.0, -141.232797
100.0, -142.28636
100.0, -142.828631
100.0, -142.610378
100.0, -141.989762
100.0, -141.28608
100.0, -139.91142
100.0, -138.514457
100.0, -136.783526
100.0, -134.751205
100.0, -132.487755
100.0, -129.944092
100.0, -127.094862
100.0, -123.935523
100.0, -120.488712
100.0, -116.754105
100.0, -112.663912
100.0, -108.390408
100.0, -103.92156
100.0, -99.247897
100.0, -94.474258
100.0, -89.671134
100.0, -84.924104
100.0, -80.308338
99.999999, -75.878584
100.0, -71.679499
100.0, -67.753688
100.0, -64.136673
100.0, -60.808247
100.0, -57.727612
100.0, -54.838422
99.999783, -52.28304
100.0, -49.909129
100.0, -47.81563
100.0, -46.013065
100.0, -44.459949
100.0, -43.102426
100.0, -41.91737
100.0, -40.908751
99.999588, -40.051258
99.999508, -39.346599
100.0, -38.846369
99.999858, -38.515727
100.0, -38.235632
100.0, -37.988543
100.0, -37.886419
100.0, -37.801069
100.0, -37.721987
100.0, -37.525726
100.0, -36.898883
100.0, -35.260675
99.999996, -31.430261
99.999989, -19.122712
```

100.0, 22.482606
100.0, 94.844759
100.0, 122.58744
99.99861, 132.733023
100.0, 137.196231
100.0, 138.977787
100.0, 139.664033
100.0, 139.656609
100.0, 139.183065
100.0, 138.442526
100.0, 137.547037
100.0, 136.576546
100.0, 135.601861
100.0, 134.675285
100.0, 133.821087
100.0, 133.03676
100.0, 132.345587
100.0, 131.780468
100.0, 131.351374
100.0, 131.126904
100.0, 131.059006
100.0, 131.159273
100.0, 131.418924
100.0, 131.874192
100.0, 132.500909
100.0, 133.294617
100.0, 134.292734
100.0, 135.530145
100.0, 137.085136
100.0, 139.023087
100.0, 141.00146
100.0, 143.76174
100.0, 147.479645
100.0, 156.373339
100.0, 0.747722
99.999994, -18.207262
99.998102, -15.600305
100.0, -11.396097
100.0, -7.855169
100.0, -4.971063
99.999914, -1.848728
100.0, 1.459119
100.0, 4.851378
100.0, 8.308167
100.0, 11.818941
100.0, 15.356981
100.0, 18.871567
100.0, 22.285835
99.998935, 25.494582
100.0, 28.362688
100.0, 30.730168
100.0, 32.426405
99.998572, 33.298452
100.0, 33.236265
100.0, 32.211772
100.0, 30.286376
100.0, 27.605847
100.0, 24.3672
100.0, 20.763412
100.0, 16.943085
100.0, 13.042265
100.0, 9.199686
100.0, 5.461704
100.0, 1.836556
100.0, -1.631536
100.0, -4.898273
100.0, -7.946003
100.0, -10.781691
100.0, -13.372326
100.0, -15.739596
100.0, -17.834294
100.0, -19.670562
100.0, -21.21365
100.0, -22.464726
100.0, -23.412506
100.0, -24.038631
100.0, -24.351516
100.0, -24.362389
100.0, -24.026437
100.0, -23.40038
100.0, -22.422741
100.0, -21.376863
100.0, -19.604398
100.0, -17.857746
100.0, -15.800266
100.0, -13.479015
100.0, -10.93416
100.0, -8.146087
100.0, -5.135199
100.0, -1.912926
100.0, 1.459882
100.0, 5.027403
100.0, 8.848628
100.0, 12.608085
100.0, 16.549325
100.0, 20.374289
100.0, 24.035229
100.0, 27.334894
100.0, 30.095657
100.0, 32.114776
100.0, 33.239747
100.0, 33.388489
100.0, 32.571326
100.0, 30.887045

100.0, 28.493556
100.0, 25.570257
100.0, 22.288403
100.0, 18.7933
100.0, 15.199895
99.998499, 11.595966
100.0, 8.036855
100.0, 4.558568
100.0, 1.165512
100.0, -2.098353
100.0, -5.162875
100.0, -7.762385
100.0, -10.477116
100.0, -12.34739
100.0, -11.999236
100.0, -24.000964
100.0, 147.386534
100.0, 145.772137
100.0, 142.149185
100.0, 139.811918
100.0, 137.966932
100.0, 136.393744
100.0, 135.005046
100.0, 133.826762
99.999978, 132.852886
100.0, 132.068574
100.0, 131.46774
100.0, 131.047594
100.0, 130.797125
100.0, 130.726124
100.0, 130.822171
100.0, 131.08098
100.0, 131.494334
100.0, 132.051595
100.0, 132.740851
100.0, 133.546452
100.0, 134.449311
100.0, 135.42028
100.0, 136.423467
100.0, 137.416124
100.0, 138.341979
100.0, 139.123516
100.0, 139.632332
100.0, 139.731568
100.0, 139.101088
100.0, 137.207045
100.0, 133.038373
100.0, 124.404035
99.999935, 97.31823
100.0, 25.462193
100.0, -17.677175
100.0, -31.105177
100.0, -35.425975
100.0, -37.349333
100.0, -37.979728
100.0, -38.14695
100.0, -38.19794
100.0, -38.323272
100.0, -38.424866
100.0, -38.579552
100.0, -38.82613
100.0, -39.20667
100.0, -39.761182
100.0, -40.488457
100.0, -41.385008
100.0, -42.451676
100.0, -43.687782
99.999257, -45.116379
100.0, -46.738595
99.99929, -48.576541
99.999281, -50.646275
100.0, -52.964023
99.999276, -55.535225
99.999259, -58.378066
99.999268, -61.459862
99.999211, -64.864552
99.999175, -68.537498
99.999197, -72.452697
99.999345, -76.631801
99.9996, -81.061523
99.999816, -85.645598
99.999928, -90.345082
99.999974, -95.123353
99.999991, -99.857798
99.999997, -104.502544
99.999999, -109.008811
100.0, -113.324921
100.0, -117.345214
100.0, -121.12764
100.0, -124.597781
100.0, -127.812958
100.0, -130.720953
100.0, -133.314139
100.0, -135.672896
100.0, -137.731815
100.0, -139.506318
100.0, -141.000265
100.0, -142.1897
100.0, -143.084349
100.0, -143.629246
100.0, -143.80431
100.0, -143.460755
100.0, -142.235327
100.0, -139.792134

```
100.0, -135.50352
100.0, -128.273712
100.0, -116.480944
100.0, -100.188597
100.0, -83.3766
100.0, -69.540656
100.0, -59.255389
100.0, -51.627231
100.0, -45.673366
100.0, -40.721137
99.999999, -36.503258
99.999997, -32.556429
99.999979, -28.958853
99.999845, -25.446275
99.999096, -21.757008
99.99868, -18.071416
99.999505, -13.956322
100.0, -9.294006
100.0, -3.697378
##END=
```

S7 Broadband refocusing pulse shape used (^{1}H)

Broadband universal rotation 180°pulse shape (BURBOP-180) for a bandwidth of 5 kHz, an rf-amplitude $\nu_{\rm rf}$ = 20 kHz, B_1-field compensation of ±30%, and a pulse length t_p of 300 μs in Bruker Topspin shape format (amplitude, phase). Quality factor of the pulse shape was >0.99999 . Please note that it is *not* J-compensated like the BUBI (Ehni and Luy, 2013) or BUBU (Ehni et al., 2022) pulse sandwiches. The pulses, however, are very short and unwanted effects within the bilinear rotations will be small. To save space, we present it in two-column format.

```
##TITLE= BURBOP180x_300us_BW5k_RF20k_pm30_99999.brk
##JCAMP-DX=
##DATA TYPE= Shape Data
##OWNER= vm1188
##DATE= 2025-10-14
##Max_Amplitude= 20000.0
##PULSE_LENGTH= 1.0 us
##NPOINTS= 300
##XYPOINTS= (XY..XY)
99.999065, 172.678265
99.998529, 151.376823
99.99993, 131.248288
99.999566, 113.69846
99.998269, 99.363945
99.998695, 88.282618
99.999532, 80.214311
99.998426, 74.861714
99.998369, 71.972405
99.998891, 71.369894
99.998876, 72.924567
99.999936, 76.505131
99.999817, 81.889482
99.999889, 88.719232
99.999986, 96.496808
99.998742, 104.673167
99.998827, 112.756948
99.999297, 120.362768
99.999083, 127.215939
99.9997, 133.17078
99.999192, 138.187284
99.999974, 142.292313
99.999985, 145.535881
99.999566, 147.938783
100.0, 149.48643
99.999962, 150.136686
99.999695, 149.833648
100.0, 148.513409
99.999999, 146.113831
99.999452, 142.569666
99.999994, 137.883136
99.999652, 132.074755
99.999859, 125.305957
99.999531, 117.847385
99.999673, 110.090731
100.0, 102.494654
100.0, 95.522146
99.999762, 89.512262
99.999596, 84.474322
99.998967, 80.24602
99.999998, 76.959932
99.999997, 74.789848
99.999488, 73.550887
100.0, 72.930683
99.999999, 72.850721
99.999981, 73.371856
100.0, 74.441799
99.999722, 75.954341
100.0, 77.826189
100.0, 80.019688
100.0, 82.506719
100.0, 85.245508
99.999721, 88.195596
100.0, 91.317321
100.0, 94.573116
99.999954, 97.929
100.0, 101.355322
100.0, 104.808155
99.999561, 108.239118
99.999563, 111.592733
100.0, 114.822975
100.0, 117.959385
100.0, 120.994806
100.0, 123.776012
100.0, 126.225054
100.0, 128.403382
99.999456, 130.168553
99.999937, 131.48218
100.0, 132.361485
100.0, 132.742733
100.0, 132.463799
100.0, 131.597783
100.0, 129.903137
```

99.999999, 127.766542
99.999999, 125.69388
100.0, 126.723428
100.0, 139.809244
100.0, 178.732641
99.999999, -141.039409
99.999279, -117.956966
99.998973, -105.710323
99.999994, -99.030287
100.0, -96.193802
100.0, -95.661169
99.999989, -95.896559
99.999283, -96.329485
99.999871, -96.865216
100.0, -97.405097
100.0, -97.820026
100.0, -98.029848
99.998694, -98.00918
100.0, -97.750765
100.0, -97.238426
99.999871, -96.438307
99.997831, -95.286857
99.998356, -93.774314
99.996735, -91.639294
100.0, -87.276682
99.999668, -80.585308
99.999994, 61.04477
99.999246, 128.517653
99.997638, 129.029567
99.999984, 131.016937
100.0, 130.928942
100.0, 130.698377
99.999992, 130.223598
99.9995, 129.335271
99.999973, 128.017484
100.0, 126.234482
100.0, 123.965511
100.0, 121.204251
100.0, 117.949289
100.0, 114.209888
100.0, 110.018822
100.0, 105.437762
100.0, 100.548621
100.0, 95.440623
99.999152, 90.205824
100.0, 84.944759
100.0, 79.753875
99.999224, 74.720066
100.0, 69.915942
99.999244, 65.390117
100.0, 61.177267
100.0, 57.298051
100.0, 53.75985
100.0, 50.56323
100.0, 47.70215
100.0, 45.175701
100.0, 42.979485
100.0, 41.089557
100.0, 39.537838
100.0, 38.306901
100.0, 37.285183
99.999994, 36.589304
99.998735, 36.174258
99.998613, 35.937031
99.999684, 35.95352
99.998486, 36.358413
100.0, 37.016611
100.0, 37.633217
100.0, 38.309147
100.0, 39.918094
100.0, 39.524185
100.0, 40.125344
99.999999, 36.184884
100.0, 33.106372
100.0, 28.333373
100.0, 8.625448
100.0, -12.892778
100.0, -26.046628
100.0, -16.556865
99.999279, 7.138899
100.0, 29.868181
100.0, 41.831938
100.0, 46.706733
100.0, 47.346605
100.0, 44.155877
99.999998, 40.718763
99.999998, 38.313821
100.0, 36.283088
100.0, 34.64414
99.999997, 33.48571
99.999982, 32.77833
100.0, 32.505337
100.0, 32.680152
100.0, 33.231148
100.0, 34.081444
100.0, 35.263454
99.999302, 36.832394
99.999954, 38.768249
100.0, 41.023474
100.0, 43.588352
100.0, 46.457923
100.0, 49.606645
100.0, 53.017356
100.0, 56.705459

99.999411, 60.701343
100.0, 65.004192
100.0, 69.557591
100.0, 74.275218
100.0, 79.085474
99.999996, 83.937139
100.0, 88.777086
100.0, 93.524265
99.999958, 98.0966
100.0, 102.467618
100.0, 106.599302
100.0, 110.401881
100.0, 113.781757
100.0, 116.690911
100.0, 119.145341
100.0, 121.226374
100.0, 122.944384
100.0, 124.336758
100.0, 125.504342
99.999491, 126.49177
100.0, 127.203167
100.0, 128.87547
100.0, 129.090422
99.999985, 134.441716
100.0, 84.414177
99.998706, -78.396463
99.999935, -88.278411
100.0, -92.676889
100.0, -95.232563
100.0, -97.335214
100.0, -98.965247
100.0, -100.205147
99.999988, -101.196618
100.0, -101.963093
100.0, -102.51407
100.0, -102.848952
100.0, -102.992286
100.0, -102.995997
100.0, -102.865198
100.0, -102.626038
100.0, -102.170966
99.99774, -102.271915
100.0, -105.290953
100.0, -111.543376
99.997322, -117.139368
99.999634, -144.613089
99.998212, 171.030553
99.99999, 130.589332
100.0, 120.327526
100.0, 121.109818
100.0, 123.601595
100.0, 124.902787
99.999469, 126.018502
100.0, 126.439314
100.0, 126.382576
100.0, 125.648511
100.0, 124.704728
100.0, 123.49168
100.0, 121.727031
100.0, 119.540051
100.0, 117.237176
100.0, 114.791066
100.0, 112.054955
99.999493, 109.046779
100.0, 105.866575
100.0, 102.582799
100.0, 99.229899
100.0, 95.834987
100.0, 92.42907
100.0, 89.048169
99.999968, 85.732298
99.999831, 82.523904
99.999974, 79.467
99.999453, 76.610637
99.999998, 74.001464
99.999436, 71.69633
99.999426, 69.765604
99.999415, 68.304479
99.999887, 67.384413
99.999391, 66.981068
99.999377, 67.086255
99.999362, 67.967609
99.999347, 69.831165
99.999331, 72.550681
99.999313, 76.215865
99.999292, 80.971306
99.999274, 86.64137
99.999261, 93.054115
99.999255, 100.051062
99.999275, 107.328912
99.999312, 114.509466
99.999365, 121.24213
99.999443, 127.248396
99.999523, 132.33074
99.999601, 136.360618
99.999669, 139.27999
99.999725, 141.106877
99.999766, 141.885865
99.999798, 141.634048
99.99982, 140.338944
99.999833, 138.040273
99.999839, 134.825107
99.999837, 130.698343
99.999827, 125.761749

```
99.999807, 119.557368
99.999774, 112.925678
99.999722, 104.987322
99.999643, 97.851548
99.999526, 89.90598
99.999368, 82.768883
99.999155, 76.620001
99.998902, 71.696247
99.998652, 68.282156
99.99845, 66.587006
99.998297, 66.791397
99.998177, 69.074325
99.997979, 73.696021
99.997614, 80.98221
99.997792, 91.498229
99.999998, 105.359399
99.998251, 122.830557
99.998528, 143.461293
99.999518, 165.723202
##END=
```

References

Ehni, S. and Luy, B.: BEBE^{tr} and BUBI: J-compensated Concurrent Shaped Pulses for ^{1}H–^{13}C Experiments, J. Magn. Reson., 232, 7–17, https://doi.org/10.1016/j.jmr.2013.04.007, 2013.

Ehni, S., Koos, M. R., Reinsperger, T., Haller, J. D., Goodwin, D. L., and Luy, B.: Concurrent J-evolving Refocusing Pulses, J. Magn. Reson., 336, 107 152, https://doi.org/10.1016/j.jmr.2022.107152, 2022.

Woordes, Y. T., Reinsperger, T., Ehni, S., and Luy, B.: Robust Bilinear Rotations, Sci. Adv., 11, eadx7094, https://doi.org/10.1126/sciadv.adx7094, 2025.
